# Foreign body in the bladder: A case report

**DOI:** 10.1016/j.ijscr.2017.02.003

**Published:** 2017-02-05

**Authors:** Kota Shimokihara, Takashi Kawahara, Yutaro Hayashi, Sohgo Tsutsumi, Daiji Takamoto, Taku Mochizuki, Yusuke Hattori, Jun-ichi Teranishi, Yasuhide Miyoshi, Yasushi Yumura, Masahiro Yao, Hiroji Uemura

**Affiliations:** a Departments of Urology and Renal Transplantation, Yokohama City University Medical Center, Yokohama, Japan; b Department of Urology, Yokohama City University Graduate School of Medicine, Yokohama, Japan

**Keywords:** Foreign body, Bladder, Higher bladder incision

## Abstract

Foreign bodies are occasionally reported in the bladder. In most cases, the foreign body is removed via the transurethral approach. A 57-year-old male patient was referred to our hospital to undergo the retrieval of a foreign body from his bladder. However, the foreign body had become severely calcified and could not be removed transurethrally. Thus, an open bladder wall incision was necessary to remove it. We herein report a case of a foreign body in the bladder that had become calcified and which was successfully removed using a higher bladder incision approach. A careful preoperative examination should be performed to detect the characteristics of the foreign body and avoid the risk of bladder wall perforation.

## Highlights

• We experienced the foreign body had become severely calcified.
• The foreign bogy was successfully removed by bladder wall incision.
• The foreign body was covered by labor.

## 1 Introduction

More than 1500 cases involving a foreign body in the bladder have been reported in Japan. In most cases, the foreign body is removed via the transurethral approach. However, the foreign body sometimes develops calcification. We herein report a case of a foreign body in the bladder that had become calcified and which was successfully removed using a higher bladder incision approach. This work has been reported in line with the SCARE criteria [1].

## 2 Case presentation

**Fig. 1:**
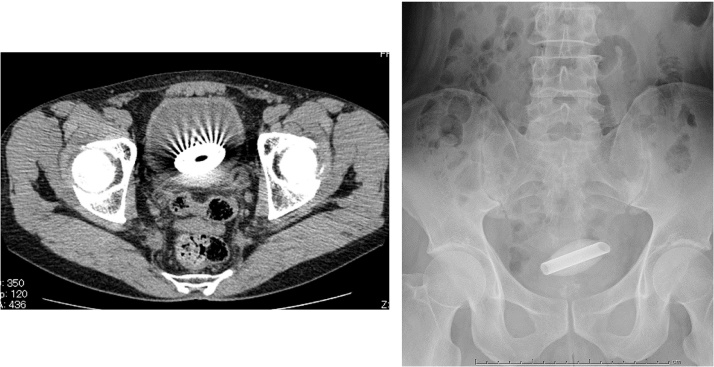
CT and KUB images of foreign body. A foreign body with an elliptical shape was found in the bladder. No mucous injury was observed.

**Fig. 2:**
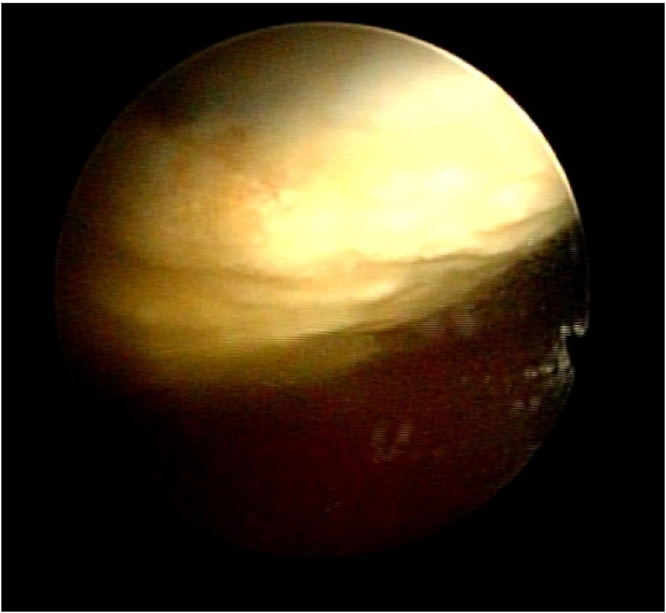
The cystoscopic findings of the foreign body. The foreign body was floating in the vesicle.

**Fig. 3:**
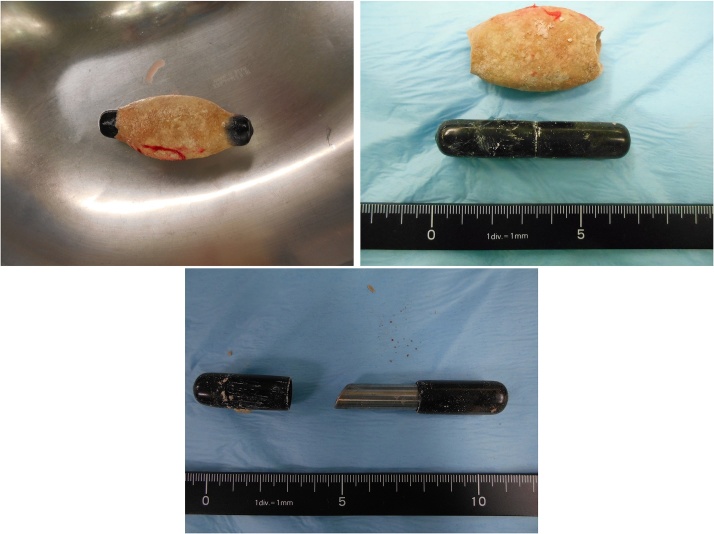
The removed foreign body in the bladder. A metallic pipe covered with elastic rubber with a smooth surface.

A 57-year-old male patient was referred to our hospital to undergo the removal of a foreign body from his bladder. He had inserted a self-made foreign body into himself several months previously [Fig. 1]. We first attempted to retrieve the foreign body via the transurethral approach; however, it had become severely calcified and could not be removed [Fig. 2]. We then performed an open bladder wall incision to successfully remove the foreign body. The removed object was a metallic tube that was covered with elastic rubber, which had a smooth surface and which had undergone calcification [Fig. 3]. At two weeks after surgery, the urethral catheter was successfully removed; the absence of leakage was confirmed by cystography.

## 3 Discussion

Cases involving foreign bodies in the bladder have been reported worldwide. In Japan, the first case was reported in 1917; since then, more than 1500 cases have been reported [2]. Foreign bodies are introduced via the transurethral approach in 60% of cases and via the trans-bladder wall approach in 30% of cases. In most recent cases, the foreign body is introduced via the transurethral approach [3]. More than 80% of foreign bodies introduced via the transurethral approach were introduced for sexual purposes, while all foreign bodies introduced via the trans-bladder approach were iatrogenic. In the present case, the patient introduced a magnetic foreign body to relieve his incontinence. Many foreign bodies have been reported, including stitches, clinical thermometers, pencils, and rubber [3]; male patients are encountered about twice as frequently as female patients. Most foreign bodies are reported in patients of 10–20 years of age; however, recently, older patients (approximately 60 years of age) have also been reported.

The main symptoms of a foreign body in the bladder include lower abdominal pain, gross hematuria, bladder irritation, and urinary tract infection [4]. Bladder stones from sometimes develop in chronic cases [5]. KUB and cystoscopy are the standard approach for diagnosing and evaluating foreign bodies in the bladder, while CT and MRI are useful in some cases.

Because antibiotics do not continuously control foreign body-related infections, it is necessary to remove the foreign body from the bladder [4]. In most cases, the transurethral approach is attempted first; if this fails, a higher bladder incision can be performed. Bladder wall perforation can occasionally occur in patients with large foreign bodies. A preoperative diagnosis of bladder wall perforation is obtained in 60% of such cases. As such, even when urologists attempt to remove a foreign body via the transurethral approach, an invasive approach may ultimately be required [6], [7]. In the present case, the foreign body was introduced transurethrally in order to control his dysuria and incontinence. Given the thick, stony covering of the foreign body, it was suspected that it had been in place for a long time. Because of the difficulty in removing this foreign body via the transurethral approach, we performed higher bladder incision.

Recently, a wide variety of foreign bodies has been reported. Urologists should be alert to this differential diagnosis when patients present with antibiotic-resistant infections and a history of bladder irritation, even if the patient denies inserting the foreign body.

A careful preoperative examination to detect the characteristics of the foreign body should be performed to avoid the risk of bladder wall perforation. When the transurethral approach is considered to be difficult, the open bladder wall incision approach should be considered immediately.

## Conflict of interest

The authors declare that they have no competing interests.

## Ethical approval

Institutional review board of Yokohama City University Medical Center approved this study (D1507018).

## Consent

We obtained written informed consent for publication. Institutional review board of Yokohama City University Medical Center approved this study (D1507018).

## Author contribution

KS and TK wrote the manuscript.

KS, TK, YH, ST, DT, TM, YH, JT, YM performed the operation.

YY, MY, HU wrote and checked the manuscript.

## Guarantor

Takashi Kawahara.
